# Expression of VP7, a Bluetongue Virus Group Specific Antigen by Viral Vectors: Analysis of the Induced Immune Responses and Evaluation of Protective Potential in Sheep

**DOI:** 10.1371/journal.pone.0111605

**Published:** 2014-11-03

**Authors:** Coraline Bouet-Cararo, Vanessa Contreras, Agathe Caruso, Sokunthea Top, Marion Szelechowski, Corinne Bergeron, Cyril Viarouge, Alexandra Desprat, Anthony Relmy, Jean-Michel Guibert, Eric Dubois, Richard Thiery, Emmanuel Bréard, Stephane Bertagnoli, Jennifer Richardson, Gilles Foucras, Gilles Meyer, Isabelle Schwartz-Cornil, Stephan Zientara, Bernard Klonjkowski

**Affiliations:** 1 UPE, ANSES, INRA, ENVA, UMR 1161 ANSES/INRA/ENVA, Maisons-Alfort, France; 2 Virologie et Immunologie Moléculaires, UR 892 INRA, Jouy-en-Josas, France; 3 INRA, UMR1225, IHAP, Université de Toulouse, INP, ENVT, Toulouse, France; 4 Centre de Physiopathologie de Toulouse Purpan, INSERM U1043, CNRS U5282, Université Paul-Sabatier, Toulouse, France; 5 Unité de pathologie des petits ruminants, ANSES, Sophia-Antipolis, France; Nanyang Technological University, Singapore

## Abstract

Bluetongue virus (BTV) is an economically important *Orbivirus* transmitted by biting midges to domestic and wild ruminants. The need for new vaccines has been highlighted by the occurrence of repeated outbreaks caused by different BTV serotypes since 1998. The major group-reactive antigen of BTV, VP7, is conserved in the 26 serotypes described so far, and its role in the induction of protective immunity has been proposed. Viral-based vectors as antigen delivery systems display considerable promise as veterinary vaccine candidates. In this paper we have evaluated the capacity of the BTV-2 serotype VP7 core protein expressed by either a non-replicative canine adenovirus type 2 (Cav-VP7 R^0^) or a leporipoxvirus (SG33-VP7), to induce immune responses in sheep. Humoral responses were elicited against VP7 in almost all animals that received the recombinant vectors. Both Cav-VP7 R^0^ and SG33-VP7 stimulated an antigen-specific CD4^+^ response and Cav-VP7 R^0^ stimulated substantial proliferation of antigen-specific CD8^+^ lymphocytes. Encouraged by the results obtained with the Cav-VP7 R^0^ vaccine vector, immunized animals were challenged with either the homologous BTV-2 or the heterologous BTV-8 serotype and viral burden in plasma was followed by real-time RT-PCR. The immune responses triggered by Cav-VP7 R^0^ were insufficient to afford protective immunity against BTV infection, despite partial protection obtained against homologous challenge. This work underscores the need to further characterize the role of BTV proteins in cross-protective immunity.

## Introduction

Bluetongue virus (BTV) is an arthropod-borne virus transmitted to ruminants by blood-sucking *Diptera* of the genus *Culicoides*. BTV is the prototype species of the genus *Orbivirus* which belongs to the family *Reoviridae* with twenty-six serotypes described so far [1]. The non-enveloped Bluetongue virion has a complex icosahedral capsid structure that packages a genome composed of 10 double-stranded (ds) RNA segments [2]. The BTV core comprises two major (VP3 and VP7) and three minor (VP1, VP4 and VP6) proteins. The outer capsid is exclusively composed of VP2 and VP5. Four additional non-structural proteins (NS1, 2, 3/3A and 4) are produced during the viral cycle. The antigenic variability of the 26 serotypes of the virus is determined by VP2. VP7, whose sequence is relatively well-conserved between isolates, is the most abundant structural protein and the major immunogenic serogroup-reactive protein [3].

To control BTV infection in domestic animals, vaccination strategies mainly rely on inactivated or live-attenuated vaccines. The inactivated vaccines have proven to be effective and economically feasible for the control of Bluetongue during the last decades [4]. Nevertheless, live attenuated vaccines are subject to criticism, since residual virulence has been reported in experimental animals as well as in the field [5], [6]. Abortions and teratological effects have also been reported [7], [8], prohibiting their use for pregnant females. Reassortment of genome segments has also been reported between two different vaccine strains in Italy [9]. For these reasons, live-attenuated vaccines tend to be replaced by safer, inactivated vaccines, which can elicit complete protection for one year in sheep after a single injection [10]. However, both inactivated and live-attenuated vaccines allow neither differentiation between infected and vaccinated animals nor broad cross-protection across serotypes. In areas where several serotypes occur, multivalent vaccines are needed to achieve efficient protection. Alternative approaches such as the use of viral-based vectors as antigen delivery systems are needed to face the unmet requirement for a broad-spectrum, effective, safe and economical vaccine strategy [11].

Poxviruses encoding VP2 and VP5 have been shown to induce a neutralizing antibody response that generally protects animals against homologous virus [12], [13]. However, in addition to the B cell response, cytotoxic T lymphocytes (CTL) play a role in protection against BTV [14]. Among BTV proteins, VP7, NS1 and VP2 contain major T-cell epitopes [15], [16] but only the VP7 protein contains CD8^+^ T-cell epitopes that are conserved amongst BTV serotypes [17]. A recombinant capripoxvirus expressing VP7 of BTV-1 has proved to confer partial protection against a heterologous BTV-3 challenge [18]. No neutralizing antibody could be detected, suggesting that cellular immunity was involved in protection against BTV. Furthermore, IFNAR (−/−) mice immunized with a cocktail of modified vaccinia Ankara vectors expressing VP2 and VP7 associated with VP5 or NS1 antigens were protected against lethal challenge with different BTV serotypes [19]–[21]. Even though the potential of such multivalent vaccines has not as yet been evaluated in target species, evidence of cross-reactive immunity in ruminants has been noted. In particular, sheep experimentally immunized with an inactivated BTV-1 vaccine were partially protected against BTV-23 challenge through a cross-reactive CTL response [22]. That provides a rationale for the development of multiserotypic vaccines against BTV.

The present study addressed the ability of the major core protein (VP7) of BTV to elicit CD4^+^ and CD8^+^ T-cell responses in sheep and to protect animals against challenge with both homologous and heterologous BTV serotypes. We generated two recombinant vectors expressing the VP7 of BTV-2, Cav-VP7 R^0^ and SG33-VP7, derived from the canine adenovirus type 2 (CAV2) and the other from *Myxomavirus* (MYXV), respectively. CAV2 is a promising antigen delivery system based on its vaccine efficacy in various animal species and lack of pre-existing immunity in non-host species [23]. Canine adenovirus vectors expressing the rabies G protein have been shown to induce high levels of protective neutralizing antibodies against rabies in all immunized sheep [24]. MYXV-derived vectors were previously shown to express recombinant antigen in several ruminant immune cell types [13], [25] and to induce a humoral immune response in sheep against a biologically relevant antigen [25]. Immunization of sheep with a MYXV-based vector expressing the VP2 protein of BTV-8 afforded substantial protection against a highly virulent homologous BTV challenge [13]. In this work, we demonstrate that both vectors were able to induce a VP7-specific CD4^+^ response whereas only Cav-VP7R^0^ stimulated a CD8^+^ T-cell response in sheep. Thereafter, sheep were immunized using Cav-VP7R^0^ and challenged with homologous and heterologous BTV serotypes. We show that the immune responses elicited against VP7 in sheep were not sufficient to significantly protect animals against BTV infection.

## Materials and Methods

### Cells

Dog kidney cells expressing the CAV2 E1 region (DK-E1) [23] were grown as monolayers in Dulbecco's modified Eagle's medium supplemented with 10% fetal calf serum, 100 IU of penicillin per ml, and 100 µg of streptomycin per ml. Baby Hamster Kidney cells (BHK, ATCC CRL-6281) were grown as monolayers in minimum essential medium, (MEM) supplemented with 5% fetal calf serum, 100 IU of penicillin per ml, 100 µg of streptomycin per ml, and 1% L-glutamine. Rabbit kidney cells (RK13, ATCC CCL-37), CHO cells expressing the human coxsackievirus/adenovirus receptor (CHO-CAR) [26] and Vero cells (ATCC CCL-81) were grown as described for DK-E1 cells.

### Vectors

#### Generation of the Cav-VP7 R0 vector

Following isolation of RNA from BHK cells infected with a Corsican strain of BTV serotype 2 [27], the VP7 gene (Genbank accession number AF 346302) was amplified by RT-PCR. The open reading frame (ORF) of VP7 was ligated into the cloning plasmid pCR2.1 by using commercially available reagents (OneStep RT-PCR kit, Qiagen and TA cloning Kit, Invitrogen, respectively). The VP7 ORF of the BTV serotype 2 was subcloned into a CAV2-shuttle vector, within an expression cassette under the control of the CMV promoter; a detailed description of the final plasmid is available upon request. The VP7 gene was entirely sequenced in both directions. This construct was used to generate the pCav-VP7 R^0^ genome by homologous recombination in BJ 5183 bacteria [28], as previously described [29]. An E1-deleted Cav-VP7 R^0^ vector expressing the VP7 of BTV-2 was then rescued by transfection of DK-E1 cells with the recombinant genome pCav-VP7 R^0^. Cav-G R^0^ has been described [24] and is isogenic to Cav-VP7 R^0^ except that the transgene encodes the rabies glycoprotein. Vectors were amplified in DK-E1 cells, purified by double banding on CsCl gradient and titrated by end-point dilution as previously described [30]. Infectious titers were expressed as TCID_50_ ml^−1^.

#### Generation of the SG33-VP7 vector

The recombinant myxomavirus SG33-VP7, expressing the VP7 ORF under the control of the strong early/late vaccinia virus P7.5 promoter, was derived from SG33, the attenuated vaccine strain of MYXV. The donor plasmid pAT-VP7 was derived from pAT-GPT, containing the M009L and M012L sequences of SG33 required for homologous recombination and the Ecogpt selection cassette [25]. Briefly, the VP7 gene was cloned into the pAT-GPT plasmid between the M009L and M012L sequences, yielding the donor plasmid pAT-VP7. The SG33-VP7 vector was then generated by homologous recombination within the SG33 M009L and M012L genes between the donor plasmid and SG33. Recombinant viruses were selected using mycophenolic acid [31]. Five sequential rounds of plaque purification followed by end-point dilution were performed to purify recombinant from parental viruses. For each round, the purity was confirmed by PCR for the absence of parental SG33 M011L and M010L genes and for the presence of the recombinant *L2* or *M5* genes (data not shown). SG33-VP7 was propagated in RK13 cells grown in OptiMEM supplemented with 2% FCS and antibiotics as above. Titration of recombinant SG33 viruses was performed as previously described [31].

### Transgene expression

To confirm expression of VP7 protein, CHO-CAR cells cultivated in 96-well plates were transduced with 1 TCID_50_ per cell of Cav-VP7 R^0^. Two days after transduction, cells were fixed with cold acetone:ethanol (4∶1; v/v). Staining was performed by using anti-VP7 monoclonal antibody (1/500; kindly provided by ID Vet, Montpellier, France), followed by Alexa Fluor 546-coupled anti-mouse IgG (1/1000; Invitrogen). Labeling was observed with a fluorescent microscope (Nikon, eclipse TE300). Non-transduced CHO-CAR cells were used as a negative control. The expression of VP7 by SG33-VP7 was tested in RK13 cells using multichamber slide flasks. Cells were infected with recombinant virus at an m.o.i. of 1, then fixed 24 hours later with 4% (w/v) paraformaldehyde in PBS and permeabilized with 0.1% (v/v) Triton X-100 in PBS. Primary staining was performed using a rabbit anti-VP7 hyperimmune serum and secondary staining was performed with FITC-coupled swine anti-rabbit IgG (1/200; Dako). Samples were observed by using a confocal LSM Olympus microscope.

### Vaccination trials

Animal experiments were performed following the recommendations of the “Charte nationale portant sur l′éthique en expérimentation animale” established by the “Comité national de réflexion éthique sur l′expérimentation animale, Ministère de l′Enseignement Supérieur et de la Recherche et Ministère de l′Agriculture et de la Pêche”. The protocol was approved by the “Comité d′éthique sur l′expérimentation animale” of the” Ecole Nationale Vétérinaire d′Alfort” (Permit number: 2009/09). All sheep used for these experiments were purchased from herds free of BTV infection, and this status was assessed by ELISA performed on sera prepared at Day 0, prior to immunization.

In a first experiment, two-year-old female Préalpes sheep were purchased from the experimental breeding of Brouessy (INRA), and randomly assigned to two groups of 3 animals to be inoculated with either Cav-VP7 R^0^ or, as a negative control, Cav-G R^0^. Sheep received two injections of 4×10^8^ TCID_50_ of CAV2 vectors in 1 ml of PBS at a 4-week-interval: half of the viral suspension was injected by the intramuscular route, and half by the subcutaneous route. Sheep were housed indoors until euthanasia and lymph node collection at day 60.

In a second experiment, 16 twelve- to twenty-four-month old Lacaune sheep were randomly assigned to two groups. Twelve sheep were immunized twice at a four-week interval, by the intradermal route with 2×10^7^ PFU of SG33-VP7. The last four control animals were not immunized. Sera were collected at D0, D28 and D60 for evaluation of the antibody response against MYXV and BTV.

In a third experiment, four- to ten-month-old male and female sheep were housed in the experimental station of Sophia-Antipolis (Anses) and randomly assigned to five groups. Two groups of 5 animals were inoculated with Cav-VP7 R^0^ and challenged with either BTV-2 or BTV-8. Two groups of 5 and 3 animals were inoculated with Cav-G R^0^ (as negative control) and challenged with BTV-2 and BTV-8, respectively. The last group of 3 sheep received only PBS and was challenged with BTV-8. Each group contained at least one male. Sheep received two injections of 4×10^8^ TCID_50_ of CAV2 vectors in 1 ml of PBS, or only PBS, at a 4-week-interval, using intramuscular and subcutaneous routes. Viral challenge was performed on day 56 by the intradermal route with wild-type strains of serotypes 2 and 8 of BTV (MERIAL SAS, Drs Hudelet and Hamers). Before viral challenge, blood samples were taken by jugular puncture every week and sera were prepared and stored at −20°C. After viral challenge, on day 56, blood samples were taken every two days until day 64 and then once a week. Total blood samples were stored at −80°C. Sheep were housed indoors in separate conventional pens and were daily monitored for health status (rectal temperature and clinical signs) for three months.

### Lymph node collection and evaluation of cell-mediated immune responses

For assessing the VP7-specific response elicited by the Cav-VP7R^0^ vector, the left lymph nodes draining the inoculation sites were excised at D60 post-priming from each immunized sheep. Lymph node cells (LNC) were prepared from the collected lymph nodes and labeled with CFSE (500 nM, Invitrogen, Cergy-Pontoise, France) as previously described [24]. LNC were cultured in X-Vivo 15 medium (Biowhittaker) supplemented with 2% FCS and 100 IU/ml penicillin and 100 µg/ml streptomycin (complete medium), either alone (basal proliferation) or with 3 µg/ml recombinant VP7 generously provided by P. Pourquier (ID-Vet). Stimulation with concanavalin A (conA, 1 µg/ml) was performed for each LNC preparation as a positive control. After 5 days of culture, cells were labeled for detection of CD4 (GC50A1 mAb, [IgM]) and CD8 (7C2 mAb, [IgG2a]) markers using appropriate isotype controls as previously described [32]. The 7-amino-actinomycin D negative (7-AAD^-^) CD4^+^ and CD8^+^ T cells that had divided were analyzed by flow cytometry on a FACSCalibur with the CELLQuest Software (200,000 events per sample, Becton Dickinson). The percent (%) of VP7-induced specific proliferation was evaluated by the [% divided T cells cultured with VP7 protein] – [% divided T cells cultured in complete medium alone]. Horizontal bars represent the means of proliferation for each group. Differences between groups were determined using an unpaired Student *T* test using the Graphpad software (La Jolla, USA). Results were considered significant if *p<*0.05.

For assessing the VP7-specific CD4^+^ T cell response elicited by SG33 vectors, blood samples were collected at D0 and D60. PBMCs were isolated and labeled with CFSE dye as described [33]. They were incubated with a complete medium containing 10% FCS and seeded into 24-well plates at a density of 3×10^6^ cells/well in the presence of either medium or recombinant virus SG33-VP7 or SG33 empty vector (m.o.i. of 1). Five days later, the cells were labeled with PE- anti-CD4- and A647-anti-CD2 antibodies (Abd Serotec, Düsseldorf, Germany) for 20 minutes at 4°C, and viability was determined using propidium iodide (BD Biosciences Pharmingen, Le Pont de Claix, France) at 1 µg/ml. Acquisition was performed using a FACScalibur flow cytometer (Becton Dickinson), and the data were analyzed with FlowJo software. A Mann-Whitney test was used for statistical analyses using Graphpad software (La Jolla, USA).

### Serological assays

#### For the Cav-VP7 R^0^ trial

The antibody response against VP7 elicited by administration of CAV2 vectors and by BTV challenge was monitored by an indirect ELISA. The anti-VP7 response was assessed against immobilized recombinant VP7 from 96-well microplates contained in the ELISA ID Screen Bluetongue Competition kit (ID VET, Montpellier, France). Plates were blocked by incubation with 100 µl PBS- 0.05% Tween containing 2% BSA and 3% milk for 45 min at 37°C. After washing, 50 µl of a 1/20 dilution of ovine sera diluted in PBS-Tween were added and incubated for 45 min at 37°C. After washing, 50 µl of a 1/8000 dilution of HRP-conjugated rabbit anti-sheep IgG (DakoCytomation) were added and incubated for 45 min at 37°C. After washing with PBS–Tween, 100 µl of DAB substrate (SIGMAFAST 3,3′-Diaminobenzidine tablets, Sigma Aldrich) were added and color development was measured by spectrophotometry at 492 nm after addition of 100 µl of 2N H_2_SO_4_. Assays were performed in duplicate, and results were expressed as means of optical density (OD). Seroconversion against VP7 is considered significant when ODs are higher than the mean value + two standard deviations observed on day 0 (OD>m+2SD).

#### For the SG33-VP7 trial

Detection of MYXV and BTV-specific antibodies in serum was carried out using indirect ELISAs. Briefly, 96-well plates were coated with SG33-VP7 (1 µg/well) or VP7 protein (1 µg/well) in PBS- 0.1% Tween-3% BSA and incubated overnight. After washing with PBS-Tween, serial dilutions of test sera were incubated for 1 hour at 37°C. Plates were washed and HRP-conjugated donkey anti-sheep IgG (1/5000, Serotec) was added for 1 hour at 37°C. After washing, substrate solution (tetramethylbenzidine at 2.4 mg/ml in citrate buffer, Sigma, France) was added for 15 minutes and color development was measured by spectrophotometry at 492 nm after addition of sulphuric acid (1N). Titers were determined by comparison with a hyperimmune serum as reference and expressed in arbitrary units. The higher point range was arbitrarily defined as 1000 units.

### Viral burden

Viral burden in plasma was determined by real-time quantitative reverse transcription PCR (RT-PCR) according to the protocol developed by Toussaint et al. [34]. RNA was extracted from 100 µl of plasma by using commercially available reagents (QIAamp Viral RNA Mini Kit, Qiagen) with an automated sample preparation system (QIAcube, Qiagen). Seven µl of extracted RNA were mixed with 0.7 µl of dimethylsulfoxide (DMSO) and heated at 95°C for 3 minutes. Each 25 µl RT-PCR mixture contained 20 µl of TaqVet Blue-Tongue Virus RT-PCR mix (LSI, Lyon, France) and 5 µl of sample or RNA standard. Reverse transcription and amplification were performed by using a 7300 Real Time PCR System (Applied Biosystems). The BTV copy number per RT-PCR mixture was evaluated by the cycle threshold (Ct)value.

### Clinical observations

Clinical examination of animals was performed daily from D3 before to D7 after each vaccination. In the third experiment, body temperature and clinical signs were recorded daily from D56 to D70 and then on D77, D84 and D90. General behavior of the animals was scored as follows: good (0); apathic (1); prostrated (2) and decubitus (3) and the most frequent clinical signs observed during Bluetongue infection were also recorded as previously described [4].

Clinical score variable was calculated by the sum of the individual daily clinical scores for each animal, divided by the number of days of clinical examination. Mean individual scores were then averaged for each group. Mortality was not taken into account in the clinical score but was indicated.

### Statistical analyses

Statistical analyses were carried out using GraphPad PRISM version 5.00 for WINDOWS to calculate mean, SD and p values (GraphPad Software, San Diego, CA).

## Results

### Expression of VP7 from recombinant vectors

**Figure 1:**
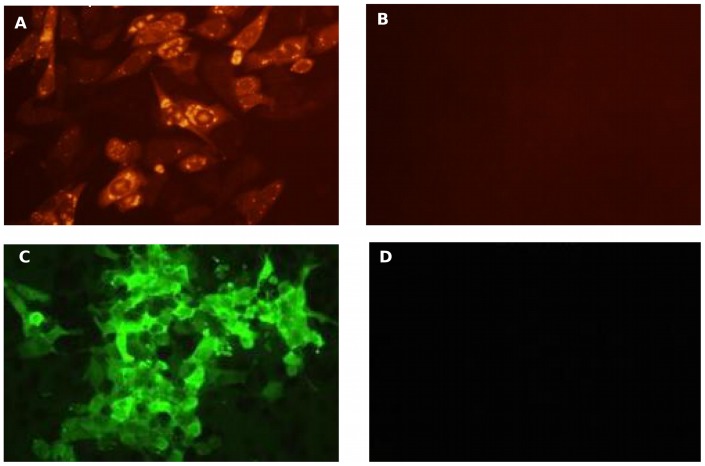
Expression of VP7 antigen. Following transduction of CHO-CAR cells with Cav-VP7 R^0^, expression of bluetongue antigen was sought and detected by immunostaining using a MAb directed against the VP7 protein (A). Following transduction of RK13 cells with SG33-VP7, VP7 antigen was labeled using an anti-VP7 hyperimmune rabbit serum (C). Non-transduced cells were used as negative control (B and D).

Recombinant vaccine vectors encoding the core VP7protein from a Corsican strain of BTV serotype 2, Cav-VP7 R^0^ and SG33-VP7, were derived from the canine adenovirus serotype 2 (CAV2) or the attenuated vaccine strain of myxomavirus, SG33, respectively. Expression of VP7 was sought by immunostaining following transduction of the CHO-CAR and RK13 cells with Cav-VP7 R^0^ or SG33-VP7, respectively. Labeling was observed in transduced cells by using antibodies directed against VP7 (Figure 1, A and C) without any expression in the control groups (Figure 1, B and D).

Following transduction of DK-E1 cells with Cav-VP7 R^0^, expression of VP7 was detected by Western blotting using serum of a sheep infected with serotype 2 of BTV (data not shown). These data confirm the expression of VP7 from both CAV2- and SG33 based vectors.

### Antibody responses in vaccinated sheep

**Figure 2:**
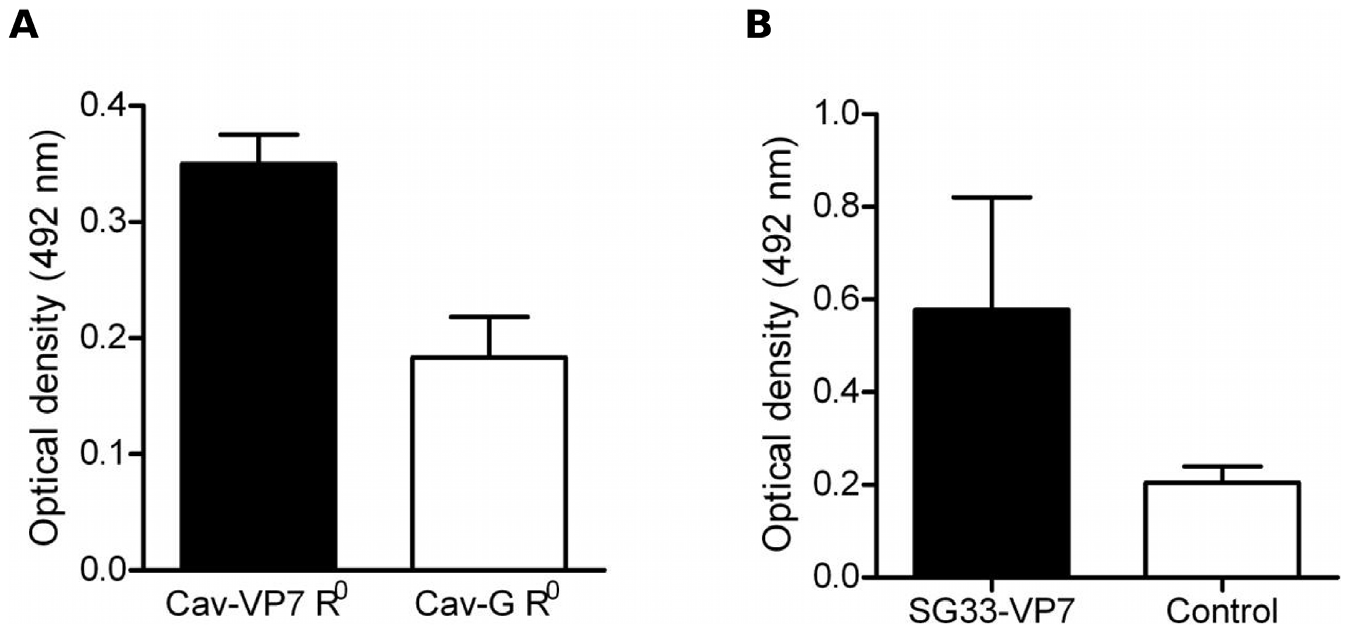
Serum antibodies induced by Cav-VP7 R^0^ and SG33-VP7 in sheep. Sheep were immunized twice with recombinant vectors. Antibody responses elicited against the VP7 protein were assessed by ELISA at day 60 following the first inoculation of Cav-VP7 R^0^ (A) or SG33-VP7 (B). Data are presented as means (with standard deviation) of individual ODs obtained for sheep inoculated with recombinant vector (black bar) or for control animals (white bar).

Sheep were immunized twice by the intramuscular and subcutaneous routes or the intradermal route with Cav-VP7 R^0^ and SG33-VP7, respectively. Cav-G R^0^ was used as a control in CAV2 trials. Ovine sera were assayed for anti-VP7 immunoglobulins by ELISA. In sheep that received recombinant vectors, antibodies directed against VP7 were detectable on day 60 after initial vaccination (average OD: 0.351 and 0.578 for Cav-G R^0^ and SG33-VP7, respectively), in contrast to control vaccinated sheep (average OD: 0.1813 and 0.205 for Cav-G R^0^ and SG33-VP7, respectively) (Figure 2). Seroconversion against VP7 was considered significant when ODs are higher than the mean value + two standard deviations observed with the control virus (OD>0.305 and OD>0.343 for Cav-G R^0^ and SG33-VP7, respectively).

### Cell-mediated responses in vaccinated sheep

**Figure 3:**
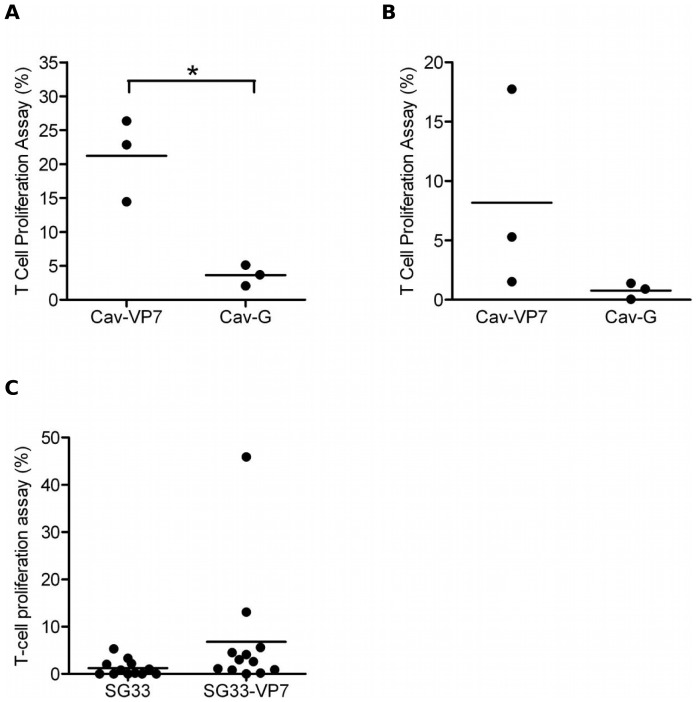
Inoculation of sheep with Cav-VP7 R^0^ and SG33-VP7 elicits T-cell responses. Sheep were immunized twice at a four-week interval with recombinant vectors and cellular immune responses were analyzed at D60. Prescapular lymph node cells were isolated and cultured in complete medium alone or with the VP7 recombinant protein. The specific proliferation was evaluated as [the % divided T cells cultured with VP7 protein] − [the % divided T cells cultured in medium alone]. (A) The percentage of CD8^+^ T cells that had divided in the absence or presence of VP7 is shown (* statistical difference between immunized groups *p* = 0.0085). (B) The percentage of CD4^+^ T cells that had divided in the absence or presence of VP7 is shown. (C) PBMCs were labeled with CFSE and cultured in complete medium alone -or with either SG33 or SG33-VP7. VP7-specific CD4^+^ T cell proliferation was evaluated as [the % divided CD4^+^ T cells cultured with recombinant vector] − [the % divided CD4^+^ T cells cultured in medium alone]. Horizontal bars represent the means of proliferation for each group.

To establish whether Cav-VP7 R^0^ and SG33-VP7 were able to induce T-cell responses against VP7, sheep were immunized twice, 4 weeks apart, with Cav-VP7 R^0^, Cav-G R^0^ or SG33-VP7. One month after the second injection, prescapular node cells (Cav experiment) or peripheral blood mononuclear cells (SG33 experiment) were harvested and the proliferative T-cell response elicited against VP7 was evaluated (Figure 3). The VP7-specific response observed in lymph node CD8^+^ T cells was significantly higher in the group vaccinated with Cav-VP7 R^0^ (average: 21.21%±3.532) than in the control group that received Cav-G R^0^ (average: 3.630%±0.8869, p = 0.0085, Figure 3A). Moreover, the VP7-specific CD4^+^ T-lymphocyte response in the draining lymph nodes tended to be higher in sheep inoculated with Cav-VP7 R^0^ (average: 8.182%±4.899) than sheep inoculated with Cav-G R^0^ (average: 0.7800%±0.3922, p = 0.2065, Figure 3B). Similarly, the CD4^+^ T-cell proliferation of PBMCs from sheep immunized with SG33-VP7 was higher in the presence of SG33-VP7 (average: 6.817%±3.701) than in the presence of the control vector SG33 (average: 1.233%±0.4865). These results suggested that immunization with SG33-VP7 and Cav-VP7 R^0^ primed a CD4^+^ T-cell response against VP7. Furthermore, data show that Cav-VP7 R^0^ vector induces CD8^+^ T-cell to proliferate when restimulated *in vitro* by the VP7 protein.

### Homologous challenge in vaccinated sheep with Cav-VP7 R^0^

**Figure 4:**
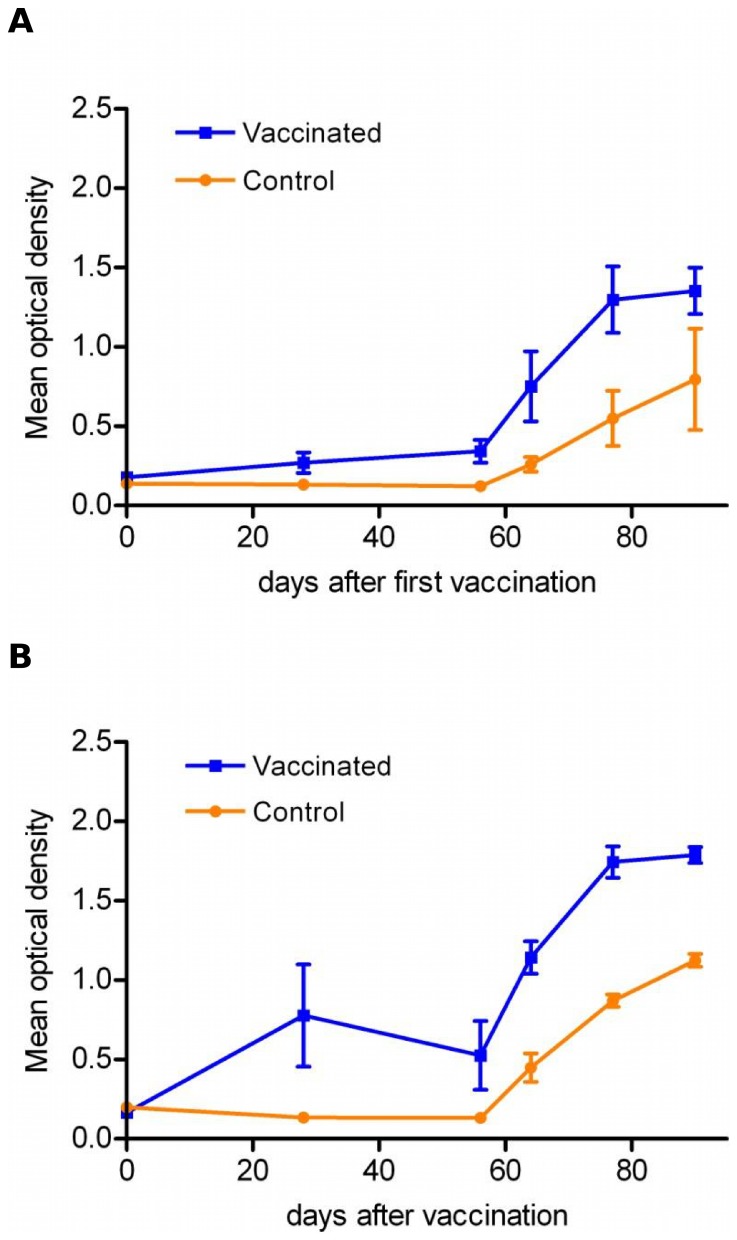
Serum antibodies induced by Cav-VP7 R^0^ or Cav-G R^0^ in sheep. Sheep received two doses of Cav-VP7 R^0^ or Cav-G R^0^ or PBS (control groups) on D0 and D28; the sheep were challenged with two wild type strains of BTV on day D56. Sera were collected at days 0, 28, 56 and every two days until day 64. Antibody responses elicited against the VP7 protein were assessed by ELISA performed on plates coated with recombinant VP7 for 1/20 dilutions of individual sera are shown, for the group challenged with BTV2 (A) and with BTV8 (B).

Since Cav-VP7 R^0^ had been shown to induce both CD4^+^ and CD8^+^ T-cell responses, its capacity to afford protection against virulent challenge was assessed in a vaccination trial. Two groups of sheep were inoculated with Cav-VP7 R^0^ according to the vaccination protocol followed for the first experiment. While control sheep remained seronegative until challenge, all vaccinated sheep seroconverted against VP7 (Figure 4). By twenty-one days after challenge with BTV-2 (Figure 4A) or BTV-8 (Figure 4B), all controls had developed a high antibody response against VP7. The level of VP7-specific antibody was also boosted in the vaccinated groups after challenge.

The ability of the Cav-VP7 R^0^ vector to inhibit BTV replication in sheep was assessed by using a BTV-specific quantitative real-time RT-PCR assay. Blood samples were used to detect BTV at days D56, D59, D62, D64, D77 and D90. Relative viral burden was expressed as the threshold cycle number (Ct) necessary for the detection of BTV genome in blood samples.

**Figure 5:**
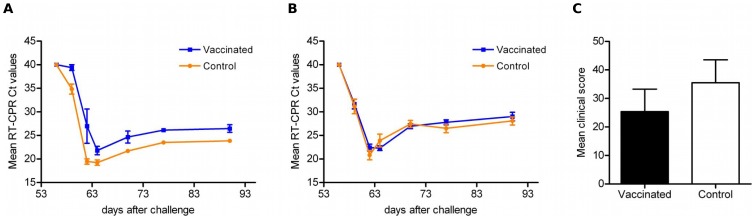
Evaluation of protection following vaccination with Cav-VP7 R^0^ and virulent challenge. (A) Evolution of mean threshold cycle (Ct) values (with standard deviation) of BTV-2 specific real-time RT-PCR (a) or BTV-8 specific real-time RT-PCR (b). (B) Mean cumulative scores (with standard deviation) after BTV-2 challenge in vaccinated (black bar) and control (white bar) groups.

For the group challenged with BTV-2 (Figure 5A), the vaccinated animals exhibited a slightly higher Ct value than those of the controls. Given that the amplification product is approximately doubled upon each PCR cycle, we noted that, from D6 to D34 after challenge, viraemia was reduced by a factor of 10 in vaccinated sheep compared to the control group. Viral loads for these sheep remained significantly lower than those observed in the control group until the end of the experiment (*p<*0.03), suggesting a partial inhibition of the BTV replication.

For the group challenged with BTV-8 (Figure 5B), no difference could be observed between viral loads of animals inoculated with Cav-VP7 R^0^ and sheep of the control group.

### Heterologous challenge in vaccinated sheep with Cav-VP7 R^0^

Clinical follow-up allowed us to establish scores for each animal from D56 (day of viral challenge) until the day of death to evaluate the protection afforded by inoculation of Cav2-VP7 R^0^. In addition, rectal temperatures were regularly recorded throughout the experiment.

During the experiment, after the virulent challenge with BTV-2, 2 of 5 sheep in the group inoculated with Cav2-VP7 R^0^ had to be euthanized due to a marked deterioration of their condition on D70. In the control group, 1 of 4 sheep was also euthanized on D70. Clinical signs typically observed during BTV infection were recorded in all groups tested with BTV-2. These included congestion of mucous membranes, generalized congestion, salivation, facial swelling, mucopurulent nasal discharge with crusting snout, anorexia, apathy, dyspnea and, in severe cases, ulcers or death.

Individual clinical scores were calculated by adding the scores obtained daily during the time of observation, from D56 to D70 (Figure 5C). For sheep #2 and #3, from the group inoculated with Cav-VP7 R^0^, clinical scores were very low (≤10). Notably, these two animals presented the lowest viral load (data not shown).

For the group challenged with BTV-8, no differences were observed between sheep inoculated with Cav-VP7 R^0^ and control groups.

## Discussion

Bluetongue virus shows substantial antigenic variability, with 26 different serotypes described to date. Vaccination has proven very effective in BTV control and conventional vaccines based on either inactivated or attenuated virus are available for many serotypes. Cross-protective immunity between BTV serotypes is very limited, so that a specific vaccine must be manufactured for each serotype. Conventional vaccines confer protection mainly through induction of neutralizing antibodies. Immunization of sheep with inactivated vaccine is not consistently associated with T-cell immunity, depending on adjuvant and/or booster dose [22], [35]. Both CD4^+^ and CD8^+^ T-cell epitopes, recognized by T-cells from infected sheep, have been identified within the VP7 antigen [16]. VP7 is a major BTV group antigen and sheep vaccinated with VP7 encoded by a capripox vector were partially protected against heterologous challenge [18]. Development of vaccines eliciting a cross-reactive T-cell response and capable of affording protection against multiple serotypes of BTV would represent a major advance in management of BTV disease.

The aim of the present study was to characterize the immune responses induced in sheep by two vaccine vectors, CAV2 and MYXV, expressing VP7. We have shown that a CAV2 vector encoding the Rabies virus glycoprotein was able to elicit a protective level of rabies neutralizing antibodies in a short period of time as well as CD4^+^ and CD8^+^ T-cell responses against the glycoprotein in all immunized sheep [24]. It is also well-established that poxviruses elicit a strong cellular immune response, and indeed an MYXV vector expressing the VP2 of BTV-8 was shown to elicit neutralizing antibody and CD4^+^ T-cell responses and afford protection in sheep [13]. Moreover, these vectors fulfil criteria for a DIVA vaccine.

Here we have shown that both non-replicative Cav-VP7 R^0^ and SG33-VP7 vectors were able to produce the conserved BTV VP7 antigen in an efficient manner and to induce both humoral and T-cell responses against VP7. These results confirm the role of the BTV VP7 protein in priming both CD4^+^ and CD8^+^ T-cell responses [16]. In agreement with a previous study [18], the vectors elicited detectable levels of VP7-binding but not neutralizing antibodies after immunization of sheep. If Cav-VP7 R^0^ vectors expressing the rabies antigen or VP7 are compared, the immune response elicited by VP7 was moderate immune, relatively delayed, and somewhat variable. Individual variability was also encountered after immunization with SG33-VP7, and only a fraction of sheep (10 of 12) developed a VP7-specific antibody response. Nevertheless, all sheep developed a strong antibody response against MYXV antigens (data not shown), attesting to an efficient vaccine delivery.

Cav-VP7 R^0^ also induced both VP7-specific CD8^+^ and CD4^+^ T-cell responses. Of note, all immunized sheep developed a CD8^+^ T-cell response, whereas the CD4^+^ T-cell proliferation was comparatively weaker and detected only in 2 of the 3 vaccinated sheep. This suggests that CAV2 vectors have a propensity for triggering potent CD8^+^ T-cell responses, as has been observed for human adenovirus-based vaccines [36]. The SG33-VP7 primed a CD4^+^ T-cell response against VP7 antigen in immunized sheep. Despite some evidence of CD8^+^ T-cell proliferation in SG33-VP7 group, no significant differences were observed between the vaccinated and control sheep, suggesting a non-specific proliferation (data not shown).

Once the immunogenicity of Cav-VP7 R^0^ had been established, its ability to afford protection against either BTV-2 or BTV-8 was assessed in sheep. Before challenge on D56, seroconversion against VP7 was observed in the majority of animals immunized with Cav-VP7 R^0^, and on D64, a booster effect due to the viral challenge was observed in all animals, demonstrating that immunity against VP7 was primed. The virological analysis performed by real-time RT-PCR showed partial albeit limited protection after homologous challenge (BTV-2). The decrease in Ct was equivalent to a 10-fold decrease in viral load compared with the average observed in the control group. Clinical examinations supported conclusions drawn from virological analysis, namely, a weak protection. For animals challenged with the heterologous BTV-8 serotype, no virological or clinical differences were observed between the group inoculated with Cav-VP7 R^0^ and the control groups, indicating that immunized sheep. We compared the VP7 amino acid sequence from BTV serotypes 2 and 8. The proteins are highly similar (97.99%), suggesting that antigenic polymorphism cannot explain the observed difference after homologous and heterologous challenge as regards viral load. In conclusion, the immune response elicited against VP7 by the Cav-VP7 R^0^ vector does not afford complete protection against homologous or heterologous challenge, despite detection of CD8^+^ and CD4^+^ T-cell responses. These results are similar to those observed with capripoxvirus vectors [37]. Absence of protection was also noted after three administrations of SG33-VP7 in sheep and subsequent challenge with BTV-8 (data not shown). These results contrast with previously published results suggesting that VP7 can elicit partial cross-protection against BTV challenge [18]. In this study, protection was only demonstrated for lethality, since all of the sheep immunized with a capripoxvirus expressing the VP7 protein from BTV-1 showed clinical signs after a heterologous challenge (BTV-3), but recovered fully from the disease in contrast to control animals. Differences between the experiments could be attributable to the BTV challenge strain or the gene transfer vectors used; of note, the capripoxvirus was replicative in ruminants and potentially expressed a higher level of VP7 protein than the non-replicative CAV2 and SG33 vectors. It is possible that the level of VP7 antigen expressed by our recombinant vectors is insufficient to prime sheep to elicit a protective immune response, as it has previously been suggested for other BTV antigens expressed in MYXV [13] or in capripoxvirus [37].

Wade Evans *et al*. were unable to detect a neutralizing antibody response after immunization of sheep with VP7. Since neither antibody dependant cell-mediated cytotoxicity (ADCC) nor complement-mediated ADCC was detected in BTV-infected sheep [38], the authors suggested that the cellular immune response was responsible for heterologous protection, although this possibility was not addressed in the study. Despite the many studies demonstrating the immunodominance of VP7 [39], we also have found that VP7-binding antibodies detected in ELISA were not associated with a protective immune response. The role of the Th1 response in protection against bluetongue disease is controversial. Adoptive transfer of lymphocytes partially protected monozygotic sheep from subsequent BTV challenge [14] and the CTL response after vaccination was suggested to protect animals against a broad spectrum of BTV serotypes [15], [22], [40]. While the protective efficacy of virus-like particles (VLPs) of BTV produced by baculoviruses has been thoroughly addressed, the protective efficacy of core-like particles (CLPs) formed by VP3 and VP7, which lack the outer membrane proteins, has not been extensively investigated [41]. A recent report described the protection afforded by VLPs and CLPs derived from a BTV-1 strain (RSA) of the western lineage against challenge with an virulent BTV-1 strain of the eastern lineage. The results indicated that an intact outer capsid consisting of VP2 and VP5 is crucial for preventing BT disease and viraemia in sheep. In contrast, CLPs containing VP7 and VP3 were unable to elicit complete protection from disease. The CLPs did, however, mitigate disease severity, suggesting that VP7 with VP3 can contribute to protection. In the present study, the VP7 protein was expressed without VP3 and its conformation might have diverged from that found in native virions. In the BTV particle, VP7 assembles as a trimer and interacts with other viral proteins, and notably with the other core protein, VP3. VP7 has been shown to modify the cellular distribution pattern of VP3, from the proteasome to perinuclear zone [42]. We do not know whether interactions between VP7 and VP3 of BTV have a major impact on antibody or cellular immune responses elicited against VP7. VP7 appeared to improve the protection afforded by a vaccine consisting of a DNA prime and a vaccinia vector boost expressing VP2 and VP5 against homologous challenge in a mouse model [19]. Recently, co-expression of VP2, VP7 and NS1 has been shown to induce neutralizing activity against the homologous challenge strain (BTV4) and CD8^+^ T-cell responses against these 3 BTV antigens and to afford cross-protection (BTV1 and BTV-8), which reinforced the presumption of a role for NS1 in multiserotype protection [20].

The humoral and cellular immunity induced in sheep by recombinant vectors expressing the VP7 encourages continued development of vaccine candidates derived from CAV2 or SG33 to protect ruminants against bluetongue. Regarding the choice of antigens to be expressed, and in view of achieving multi-serotype protection, it will be of interest to improve vaccine-elicited immunity against BTV by combining the Cav-VP7 R^0^ vector with a vector expressing other viral proteins, such as NS1 and VP2.
